# Comparing the use of Tutopatch® pericardium with Tutoplast® fascia lata in the context of PAUL® glaucoma implant surgery: an anterior segment OCT study

**DOI:** 10.1038/s41433-025-04018-3

**Published:** 2025-09-23

**Authors:** Pascal Schipper, Leonie Bourauel, Leonie Weinhold, Michael Petrak, Verena Prokosch, Frank G. Holz, Constance Liegl, Karl Mercieca

**Affiliations:** 1 University of Bonn, Department of Ophthalmology, Bonn, Germany; 2 University of Bonn, Institute for Medical Biometry, Informatics and Epidemiology, Bonn, Germany; 3 University Hospital Cologne, Department of Ophthalmology, Cologne, Germany

**Keywords:** Glaucoma, Surgery, Medical imaging

## Abstract

**Aim:**

To evaluate the use of high-resolution anterior segment optical coherence tomography (AS-OCT) for the comparison of different patch grafts and their changes in patients undergoing PAUL® Glaucoma Implant (PGI) surgery.

**Methods:**

Prospective analysis over twelve months of pericardium and fascia lata patch graft thickness of PGI surgical cases at the University Eye Hospital Bonn, Germany, from November 2021 to March 2023. In all eyes, either Tutopatch® pericardium or Tutoplast® fascia lata was used to cover the implant intra-operatively. AS-OCT examinations were performed to measure quantitative and qualitative aspects of the patch grafts before surgery, and at three, six, and twelve months after surgery.

**Results:**

50 eyes of 47 patients were included. For Tutopatch® (26 eyes) the patch material thickness directly over the tube decreased from an initial 1020 ± 302 µm to 564 ± 458 µm at twelve months after surgery whereas for Tutoplast® (24 eyes) it decreased from an initial 1575 ± 319 µm to 1380 ± 317 µm at twelve months. Tutopatch® material declined faster by −42.1 µm/month (CI: −57.2–−26.9, *p* < 0.001). Five patients in the Tutopatch® group showed tube erosion whereas none did in the Tutoplast® group. Adjusting for potential confounding variables did not substantially alter the association between the groups and patch material thickness.

**Conclusions:**

This study shows that AS-OCT is a useful method for comparing different patch materials after PGI surgery. When comparing pericardium with fascia lata, the latter evidenced a slower rate of thickness reduction and was associated with less tube exposure.

## Introduction

Glaucoma Drainage Implants (GDIs) are commonly used for secondary glaucoma (e.g. uveitic, neovascular), significant conjunctival scarring, failed bleb-forming surgery, ocular cicatricial pemphigoid (OCP), aphakic glaucoma, ICE syndrome, anterior segment dysgenesis, or as a primary intervention in open-angle glaucoma [1–4]. The most widely used GDIs include the Ahmed® glaucoma valve (AGV) and Baerveldt® glaucoma implant (BVT), while the newer PAUL® glaucoma implant (PGI) is gaining popularity.

Most GDI surgery techniques include the use of a patch material, which is placed over the tube to prevent tube erosion through the conjunctiva. A challenge in the postoperative follow-up is to identify those patients at risk of tube erosion, particularly in those who develop a rapid decrease in patch thickness. Comparative measurement of a patch material over time therefore may be particularly important here. Methods such as slit-lamp biomicroscopy, ultrasonography and magnetic resonance imaging (MRI) examination of filtering blebs and patch grafts either do not have sufficient resolution or are too expensive or cumbersome for everyday use. On the other hand, high-resolution anterior segment optical coherence tomography (AS-OCT) has been shown in some studies to be well suited for this very purpose [5, 6]. In a previous study by our group, we were able to show that AS-OCT is suitable for measuring patch materials with high reliability and, more importantly, is able to identify patients with an increased risk of tube erosion at an early stage [7]. So far, however, no study seems to have investigated a comparison of different patch materials using AS-OCT. This study demonstrates the possibility of comparing different patch materials, potentially highlighting the superiority of one patch type over another.

## Material and methods

### Patients

This study was approved by the University Hospital Bonn ethics committee, and all participants provided written informed consent. It therefore complies with the criteria of the Declaration of Helsinki.

50 patients were recruited between November 2021 and March 2023. Surgery was performed by two experienced surgeons (KM & MP) according to local protocol. High-resolution AS-OCT (Heidelberg ANTERION® Swept-Source OCT, Heidelberg Engineering GmbH, Germany) was used preoperatively and at one week, three months, six months, and twelve months after surgery to visualise and compare the two different patch materials that were used during surgery to prevent tube erosion. Retrospective clinical data were collected alongside prospective imaging data, including best corrected visual acuity (BCVA; Snellen, converted to logMAR), intraocular pressure (IOP; Goldmann applanation tonometry), as well as slit lamp and fundus examinations. Additional recorded variables included age, gender, ethnicity, glaucoma type, prior surgeries, number of eye drops and systemic conditions (e.g. diabetes, hypertension, and anticoagulant use).

### Surgical technique

The PGI was surgically implanted as described by Vallabh et al. in 2022 [8]. After a limbal incision, the subconjunctival and sub-Tenon’s space was prepared and 0.5 mg/ml mitomycin C (MMC) was applied for two minutes to the treatment area. The extraocular muscles in the chosen quadrant (usually superotemporal) were identified and the tube plate placed beneath and fixed with 9.0 nylon sutures. A 6.0 prolene intraluminal ripcord was always used and the tube placed in the anterior chamber (AC) via a two-step scleral tunnel technique. In two eyes, however, the tube had to be placed in the pars plana due to prior surgeries. The tube was secured with a scleral 9.0 suture and then covered with double-layered patch material to prevent tube erosion. Twenty-six patients who underwent surgery between November 2021 and August 2022 had received Tutopatch® pericardium (RGI Surgical, United States) and 24 patients who underwent surgery between August 2022 and March 2023 received Tutoplast® fascia lata (Bess Medizintechnik GmbH, Germany). The patch material was fixated with TISSEEL® (Baxter, United States) two-component fibrin adhesive and the conjunctiva was subsequently closed with 10.0 nylon sutures.

### Imaging protocol

AS-OCT images were recorded by trained personnel using defined parameters. Each series included 25 OCT scans of 16.0 mm × 7.5 mm at an angle of 45° or 135°, depending on the implant quadrant. One AS-OCT device was used for all examinations and measurements were conducted with Heidelberg Eye Explorer (HEYEX2). To ensure consistency, the centre between the tube’s anterior chamber entry and the beginning of the PGI plate was determined, and patch material thickness above it was measured in pixels. To calculate average patch thickness, further thickness measurements from three OCT slices above and below the tube were taken. Internal validation confirmed examiner-independent, repeatable measurements with high intraclass correlation coefficients (ICC) for both intra-rater at 0.990 and inter-rater reliability at 0.983. Bland–Altman plots (Supplementary 1) illustrate reliability analyses.

Patch thickness in micrometres (µm) was calculated using a conversion factor of 6.82, which was determined using standardised thickness measurements via Pentacam® (OCULUS Optikgeräte GmbH, Germany) and AS-OCT in relation to the non-standardised measurements in pixels, correlating 526.5 µm to 77.25 pixels.

### Statistical analysis

Statistical analysis was performed with GraphPad Prism 9.5.1. for Windows (GraphPad Software, Boston, Massachusetts, United States) and R software environment (version 4.4.0; R Foundation, Vienna, Austria). Categorical data are presented as absolute numbers and percentages, continuous data as mean (± standard deviation (SD)) and median (range).

Fisher’s exact test was used to compare nominal variables between Tutoplast® vs. Tutopatch®, including demographic factors, glaucoma type, comorbidities, lens status, number of eye drops, prior surgeries, implantation quadrant and patch material characteristics. Continuous variable distributions were assessed visually and with the Shapiro–Wilk test. The Mann–Whitney U test was applied to compare age, pre-operative BCVA (logMAR), pre-operative IOP and thickness measurements of conjunctiva and patch materials.

Linear mixed models were used to analyse factors related to a faster patch material dissolution, Tutopatch® average thickness as the target variable and the independent variables ‘Tutoplast® vs. Tutopatch®’, ‘Time since surgery’ and the interaction of the two variables with the participants ID as random intercept. Additional models adjusted for potential confounders such as age, gender, glaucoma type, previous surgery, lens status, pre-operative IOP, scleral thickness and conjunctival thickness. Results are presented by effect estimates with 95% confidence intervals (CI) and *p*-values. A linear mixed regression model was used to compare the dissolution speed of Tutopatch® and Tutoplast®. Results were considered statistically significant if the *p*-value of the interaction term was less than 0.05.

## Results

50 eyes of 47 patients who underwent PGI surgery with Tutopatch® pericardium or Tutoplast® fascia lata were included in this study, 45 of which were of European descent. Of the 26 patients with Tutopatch®, five patients developed tube exposure with the subsequent need for revision surgery. In the Tutoplast® group, no tube exposures occurred. Demographics and baseline clinical characteristics are displayed in Table 1.

**Table 1:** Patient demographics and pre-operative data.

Variable	Tutopatch® (*n* = 26; %)	Tutoplast® (*n* = 24; %)
Gender (male | female)	15 (58) | 11 (42)	14 (58) | 10 (42)
Age (mean | median | range) (years)	64.8 | 68.5 | 26–81	67.6 | 70 | 23–88
Ethnicity (Caucasian | Middle Eastern)	25 (96) | 1 (4)	23 (96) | 1 (4)
Glaucoma type:		
Primary open-angle glaucoma	12 (46)	10 (42)
Angle-closure glaucoma	1 (4)	1 (4)
Pseudoexfoliation glaucoma	2 (8)	3 (12)
Pigmentary glaucoma	1 (4)	0 (0)
Secondary glaucoma	10 (38)	10 (42)
Mean visual acuity (logMAR)	0.72 ± 0.80	0.95 ± 0.85
Mean intraocular pressure (mmHg)	24.0 ± 7.8	24.9 ± 8.5
Diabetes (yes | no)	5 (19) | 21 (81)	3 (12) | 21 (88)
Arterial hypertension (yes | no)	11 (42) | 15 (58)	13 (54) | 11 (46)
Anticoagulation (yes | no)	3 (12) | 23 (88)	3 (12) | 21 (88)
Lens status (phakic | pseudophakic | aphakic)	4 (16) | 22 (84) | 0 (0)	5 (21) | 16 (67) | 3 (12)
Post-vitrectomy (yes | no)	11 (42) | 15 (58)	7 (29) | 17 (71)
Number of eye drops (0 | 1 | 2 | 3 | 4)	0 | 0 | 2 | 10 | 14	2 | 1 | 3 | 5 | 13
Oral acetazolamide (yes | no)	8 (31) | 18 (69)	7 (29) | 17 (71)
Former glaucoma surgeries (yes | no):	13 (50) | 13 (50)	16 (33) | 8 (67)
Trabeculectomy	4 (16)	3 (12)
Cyclophotocoagulation	3 (12)	5 (21)
Canaloplasty	3 (12)	0 (0)
Trabectome	3 (12)	1 (4)
Deep Sclerectomy	0 (0)	5 (21)
XEN® Gel Stent	0 (0)	2 (8)
Tube localisation (anterior chamber | pars plana)	25 | 1	23 | 1
Implantation quadrant (superotemporal | superonasal)	22 | 4	21 | 3
Tube exposure (yes | no)	5 (19) | 21 (79)	0 (0) | 24 (100)

For Tutopatch®, conjunctival thickness changed from 272 ± 89 µm pre-operatively to 209 ± 148 µm at twelve months after surgery. The thickness of the patch material directly over the tube decreased from initially 1020 ± 302 µm to 564 ± 458 µm at twelve months after surgery. Similarly, the total thickness of the patch material changed from initially 1239 ± 307 µm to 628 ± 456 µm at twelve months after surgery, which is a reduction of 49% of the initial thickness. The Tutoplast® group showed thicker patch materials directly postoperatively, which decreased only slightly over the following twelve months. The thickness of the patch material directly over the tube decreased from initially 1575 ± 319 µm to 1380 ± 317 µm at twelve months after surgery. Total thickness of the patch material changed from initially 1670 ± 275 µm to 1517 ± 328 µm at twelve months after surgery, which equates to a reduction of 9%.

**Fig. 1:**
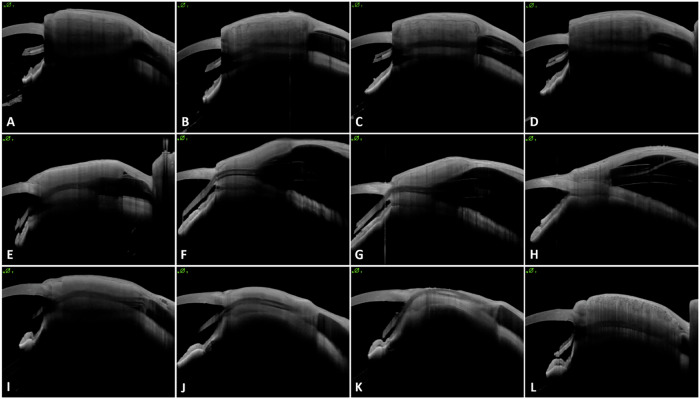
Exemplary visualisation of patch material thickness development. Visualisation of the thickness development of Tutopatch® and Tutoplast® over 12 months with repetitive high-resolution AS-OCT imaging. Representative imaging of an eye with Tutoplast® patch graft material (**A**–**D**). From directly post-operatively (**A**), to 3 months (**B**), 6 months (**C**), and to 12 months after surgery (**D**), the patch material thins out slightly. No tube exposure was detected. For Tutopatch® without tube erosion (**E**–**H**), a clear thinning of the patch material could still be observed over the course of 12 months (post-operatively (**E**), 3 months (**F**), 6 months (**G**), 12 months (**H**)). However, the patch thickness decreases early in eyes with later tube erosion – as displayed in (**I**–**L**). After 6 months (**K**), a tube exposure was detected, and the patient scheduled for revision surgery with a Tutoplast® graft (**L**).

**Fig. 2:**
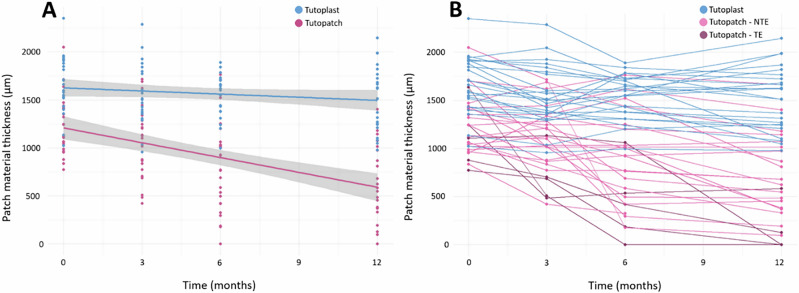
Patch material thickness development. Development of the thickness of the patch materials over time (**A**). The averaged thickness of the patch material shows a significantly faster decrease in the Tutopatch® than in the Tutoplast® eyes with a decrease rate of 42.1 µm/month. Development of the thickness of each individual patch graft (**B**). It was found that some of the eroded Tutopatch® grafts were among the thinner tissues with a thickness of less than 1000 µm, but that this was not the sole reason for later erosion. Also, not all eroded patches showed an early strong reduction in thickness. NTE no tube exposure, TE tube exposure.

Tutopatch® thickness was already significantly lower than that of Tutoplast® immediately after implantation and decreased significantly more over the course of twelve months - even in the absence of tube exposure (*p* < 0.001). Tutoplast® material thinned only slightly over the same period. Figure 1 demonstrates the reduction in thickness of the patch materials over twelve months using AS-OCT images of three patients as an example - one with Tutoplast® graft, one with Tutopatch® and tube exposure and one with Tutopatch® without exposure, while Fig. 2 shows the decrease in thickness of patch material for all patients.

A linear mixed model comparing decrease of patch thickness between Tutoplast® (−10.82 µm/month) and Tutopatch® (−52.88 µm/month), estimated that the latter declined faster by −42.1 µm/month (CI: −57.2 – −26.9, *p* < 0.001) over the first 12 months. Adjusting for potential confounding variables did not substantially alter the association between groups and thickness of patch material (age: *p* = 0.376; gender: *p* = 0.73; glaucoma subtype: *p* = 0.77; previous glaucoma surgery: *p* = 0.19; lens status: *p* = 0.30; pre-operative IOP: *p* = 0.92; pre-operative scleral thickness: *p* = 0.17). A further subgroup analysis was not possible due to the absence of tube exposure in the Tutoplast® group and only five patch erosions in the Tutopatch® group.

Twenty eyes with Tutopatch® graft showed a thin layer of fluid between the individual sheets of patch material directly after surgery, while this was the case in only eight eyes with Tutoplast®. The frequency of this decreased in both groups, with only one eye with Tutopatch® and four with Tutoplast® showing fluid at the twelve-month mark. Patch material that was exceeding the limbus was observed in 11 eyes with Tutopatch® and 16 eyes with Tutoplast® directly after surgery. After twelve months, this was seen in only one eye with Tutopatch® and seven eyes with Tutoplast®. Table 2 shows more patch material characteristics and measurements, including conjunctival thickness which decreased from 352 ± 45 µm pre-operatively to 236 ± 105 µm at twelve months after surgery. However, the conjunctival thickness was not identified as a confounding factor (*p* = 0.832). Supplementary 2 shows the patch material characteristics and measurements at 3 and 6 months.

**Table 2:** Pre- and post-operative data and patch graft measurements for Tutopatch® and Tutoplast®.

Variable	Tutopatch® (*n* = 26)	Tutoplast® (*n* = 24)
Pre-operative:		
Mean conjunctival thickness (µm)	272 ± 89	275 ± 97
Post-operative:		
Mean conjunctival thickness (µm)	304 ± 96	352 ± 45
Mean patch material thickness above the tube (µm)	1020 ± 302	1575 ± 319
Mean patch material thickness superior of the tube (µm)	1251 ± 296	1713 ± 294
Mean patch material thickness inferior of the tube (µm)	1288 ± 348	1723 ± 250
Mean overall patch material thickness (µm)	1239 ± 307	1670 ± 275
Fluid layer between the patch material layers (yes | no)	20 | 6	8 | 16
Patch material exceeding the limbus (yes | no)	11 | 15	16 | 8
1 year after surgery:		
Mean conjunctival thickness (µm)	209 ± 148	236 ± 105
Mean patch material thickness above the tube (µm)	564 ± 458	1380 ± 317
Mean patch material thickness superior of the tube (µm)	659 ± 447	1558 ± 343
Mean patch material thickness inferior of the tube (µm)	661 ± 482	1549 ± 489
Mean overall patch material thickness (µm)	628 ± 456	1517 ± 328
Fluid layer between the patch material layers (yes | no)	1 | 21	4 | 19
Patch material exceeding the limbus (yes | no)	1 | 21	7 | 16

Both groups were analysed for differences in several variables in an exploratory analysis. Table 3 displays a detailed comparison of gender, ethnicity, glaucoma type, other eye diseases, diabetes, arterial hypertension, use of anticoagulants, lens status, post-vitrectomy status, number of pre-operative eye drops, status of previous glaucoma surgery, tube localisation and implantation quadrant as well as conjunctival and patch material thickness. No differences were found between the Tutopatch® and Tutoplast® groups regarding patient characteristics. Neither pre-operative IOP and visual acuity nor the conjunctival thickness were significantly different. Detailed information on the individual parameters can be found in Table 3. Additionally, Supplementary 3 shows the group statistics and comparison of patients at 3 and 6 months.

**Table 3:** Group statistics and comparison of patients with Tutoplast® and Tutopatch®-patients with and without tube exposure.

Variable	Tutopatch® - No tube exposure (*n* = 21)	Tutopatch® - Tube exposure (*n* = 5)	Tutoplast® (*n* = 24)	*P*-value
Gender (male | female)	14 | 7	1 | 4	14 | 10	*NTE/TE:* 0.128^a^ *Patch/Plast:* 1.000^a^
Ethnicity (Caucasian | other)	20 | 1	5 | 0	23 | 1	*NTE/TE:* 1.000^a^ *Patch/Plast:* 1.000^a^
Age (years)	64.2 ± 13.3	67.4 ± 12.9	67.6 ± 15.3	*NTE/TE:* 0.741^b^ *Patch/Plast:* 0.356^b^
Glaucoma type (POAG | ACG | XFG | PG | SG)	11 | 1 | 1 | 0 | 8	1 | 0 | 1 | 1 | 2	10 | 1 | 3 | 0 | 10	*NTE/TE:* 0.173^a^ *Patch/Plast:* 0.9589^a^
Concurrent eye diseases (e.g. uveitis, high myopia, retinal vein occlusion) (yes | no)	15 | 6	2 | 3	17 | 7	*NTE/TE:* 0.302^a^ *Patch/Plast:* 0.767^a^
Diabetes (yes | no)	4 | 17	1 | 4	3 | 21	*NTE/TE:* 1.000^a^ *Patch/Plast:* 0.702^a^
Arterial hypertension (yes | no)	8 | 13	3 | 2	13 | 11	*NTE/TE:* 0.620^a^ *Patch/Plast:* 0.572^a^
Anticoagulation (yes | no)	1 | 20	2 | 3	3 | 21	*NTE/TE:* 0.085^a^ *Patch/Plast:* 1.000^a^
Lens status (phakic | pseudophakic | aphakic)	1 | 20 | 0	3 | 2 | 0	5 | 16 | 3	*NTE/TE:* 0.014^a^ *Patch/Plast:* 0.140^a^
Post-vitrectomy (yes | no)	11 | 10	0 | 5	7 | 17	*NTE/TE:* 0.053^a^ *Patch/Plast:* 0.388^a^
Former glaucoma surgery (yes | no)	10 | 11	3 | 2	16 | 8	*NTE/TE:* 1.000^a^ *Patch/Plast:* 0.265^a^
Number of eye drops (0 | 1 | 2 | 3 | 4)	0 | 0 | 2 | 6 | 13	0 | 0 | 0 | 4 | 1	2 | 1 | 3 | 5 | 13	*NTE/TE:* 0.147^a^ *Patch/Plast:* 0.324^a^
Pre-operative:				
Visual acuity (logMAR)	0.82 ± 0.86	0.32 ± 0.19	0.87 ± 0.79	*NTE/TE:* 0.603^b^ *Patch/Plast:* 0.222^b^
Intraocular pressure (mmHg)	23.5 ± 7.9	26.0 ± 8.0	25.3 ± 8.5	*NTE/TE:* 0.622^b^ *Patch/Plast:* 0.849^b^
Conjunctival thickness (µm)	276 ± 99	252 ± 28	275 ± 97	*NTE/TE:* 0.987^b^ *Patch/Plast:* 0.676^b^
Post-operative:				
Tube localisation (anterior chamber | pars plana)	20 | 1	5 | 0	23 | 1	*NTE/TE:* 1.000^a^ *Patch/Plast:* 1.000^a^
Implantation quadrant (superotemporal | superonasal)	17 | 4	5 | 0	21 | 3	*NTE/TE:* 0.555^a^ *Patch/Plast:* 1.000^a^
Conjunctival thickness (µm)	327 ± 83	207 ± 95	352 ± 45	*NTE/TE:* 0.024^b^ *Patch/Plast:* 0.115^b^
Mean patch material thickness above the tube (µm)	1232 ± 294	1077 ± 338	1575 ± 339	*NTE/TE:* 0.476^b^ *Patch/Plast:* 0.0003^b^
Mean overall patch material thickness (µm)	1265 ± 301	1129 ± 340	1670 ± 297	*NTE/TE:* 0.380^b^ *Patch/Plast:* < 0.0001^b^
12 months after surgery:				
Conjunctival thickness (µm)	259 ± 119	5 ± 12	236 ± 105	*NTE/TE:* 0.0008^b^ *Patch/Plast:* 0.48^b^
Mean patch material thickness above the tube (µm)	682 ± 427	90 ± 201	1381 ± 317	*NTE/TE:* 0.0057^b^ *Patch/Plast:* < 0.0001^b^
Mean overall patch material thickness (µm)	749 ± 414	142 ± 253	1517 ± 328	*NTE/TE:* 0.0059^b^ *Patch/Plast:* < 0.0001^b^

For numerical variables Mann–Whitney U test was used and for ordinal and categorical variables Fisher’s exact test was performed.

*NTE* ‘no tube exposure’, *TE* ‘tube exposure’, *POAG* primary open-angle glaucoma, *ACG* angle-closure glaucoma, *XFG* pseudoexfoliation glaucoma, *PG* pigmentary glaucoma, *SG* secondary glaucoma.

^a^Fisher’s exact test (Freeman–Halton extension if more than 2 × 2 contingency table).

^b^Mann–Whitney U test.

## Discussion

Tube erosion after GDI surgery is a rather uncommon, but serious complication with the need for subsequent surgical revision. In some cases, GDI explantation may eventually become necessary due to persisting exposure and the risk of infection. It is essential to recognise possible erosions early, since surgical revision is easier and potentially more successful if done in the earlier stages of this process. Early revisions may also prevent infection related to tube erosion with the subsequent need for more surgeries and potential GDI failure.

As previously shown by our group and others, AS-OCT has proven to be a reproducible and easily accessible modality to visualise patch grafts and their thickness after surgery [7]. In this study, we compared the use of Tutopatch® pericardium with Tutoplast® fascia lata as patch grafts. We showed that eyes with Tutoplast® did not develop any erosion whilst 5 of 26 eyes (19.2%) with Tutopatch® eroded during a follow-up period of twelve months in our study cohort. Tutoplast® showed thicker patch material directly postoperatively, which decreased only slightly over the following twelve months while Tutopatch® was already thinner directly after surgery showing a fast and more extensive decrease of material (Fig. 2). To the best of our knowledge, there are only two other studies analysing postoperative patch graft changes using AS-OCT. De Luna et al. investigated the outcome of gamma-irradiated sterile cornea (GISC) patch grafts and were the first to utilise AS-OCT to measure graft thickness. They included Ahmed FP7 and S-2 and Baerveldt 250-mm^2^ or 350-mm^2^ as GDI and showed that 16.6% of eyes did not have a detectable graft at a certain point after surgery. Their overall rate of graft thinning was 60 µm/year, with a follow-up time of 1.70 years (range 1 day to 6 years) [5]. Our study on the other hand only included data up to one year, with more long-term data currently being collected. Another study which used AS-OCT to look at GDI patch grafts is from Akbas et al. [6] which evaluated changes in pericardium patch graft thickness related to the AGV. This showed progressive post-operative patch reduction within twelve months, which was similar to our own findings with pericardium grafts. Tutopatch® thickness directly over the tube decreased from initially 1020 ± 302 µm to 564 ± 458 µm twelve months after surgery in our study. Akbas et al. described that the reduction rate was higher centrally over the tube (21.2% and 34.8% for 1–6 months and 6–12 months, respectively) whilst it was lower peripherally (3.5% and 5.1%, respectively), contributing the finding to higher mechanical forces occurring centrally. In our study, the mean thickness was lower over the tube (564 µm ± 458 µm) whilst it was 659 µm ± 447 µm superior and 661 µm ± 482 µm below the tube at twelve months after surgery. There seems to be a higher risk of exposure in the central area of the patch graft directly over the tube, possibly due to greater exposure to mechanical stress.

Reported rates of tube exposure tend to be lower than those described for Tutopatch® (19.2%) in our study. Different materials have been proposed as patch grafts, including pericardium [9], fascia lata [10], sclera [11], dura, amniotic membrane [12] and cornea [13]. Raviv et al. were one of the first to report on the use of pericardial patch grafts in glaucoma implant surgery in 1998 [9], with none of the eyes in their cohort having erosion. However, five out of 54 eyes were noted to have asymptomatic thinning. Smith et al. compared donor sclera, dura and pericardium patch grafts in 64 eyes with at least 24 months-follow-up and concluded that no material was more prone to melting than another [14]. Van Hoefen Wijsard et al. compared pericardium and donor sclera, showing that tube exposure was more common in the pericardium group with similar rates to our study (13.6%) [15]. Al-Beishri et al. reported a cumulative incidence of tube exposure of 6.3% (0.9% per year) for 53 cases among 836 patients [16]. They included different patch grafts in their study and the use of a scleral patch was associated with the lowest risk. De Luna et al. described an odds ratio of undetectable grafts per year after AGV surgery of 2.1 (95% CI, 1.5–3.0; *p* < 0.001) [5]. Pan et al. stated a rate of tube exposure in ten out of 319 eyes (3.13%) for GISC patch grafts after a mean follow-up of 15.4 months [17]. One potential explanation for the discrepancy in exposure rates may lie in the differing patient populations. For instance, in the study by Van Hoefen Wijsard et al. [15], only 12 out of 244 patients (4.9%) had a history of vitreoretinal surgery - a factor influencing conjunctival vulnerability. In contrast, 18 of the 50 patients in our study (36.0%) had previously undergone such procedures.

An alternative to using patch grafts is the creation of long scleral tunnels [18]. Kugu et al. compared the use of a pericardium with a scleral tunnel technique and showed that long scleral tunnels may be beneficial in preventing conjunctival tube exposure in AGV implantation surgery [19]. Some also use a partial-thickness scleral autograft as another option. It would be interesting to evaluate these tunnels with AS-OCT for scleral thinning over time, but these techniques are not routinely used in our hospital.

Identifying risk factors for tube erosions could help prevent their occurrence and to decide individually which patch graft is best suited for which patient. We performed a regression analysis to possibly identify risk factors for tube exposure, but did not find any significant ones. As this could be related to the small number of eyes with erosions, we also performed another analysis to look for factors influencing thinning rates of these two patch grafts. The latter also did not show any significant risk factors associated with faster and more extensive thinning. On the other hand, other studies have identified risk factors: Al-Beishri et al. described prior ocular surgery, female sex, and older age as possible risk factors [16]. In their study with 836 patients, the use of a scleral patch was potentially protective. Chaku et al. identified younger age and inflammation as additional risk factors when using irradiated donor pericardium [20]. De Luna et al., on the other hand, did not identify risk factors such as age or type of AGV but observed a progressive thinning of corneal grafts over time (−60 µm per year) [5]. Similar findings to De Luna et al. were found in our study showing that the patch graft thickness decreased over time (Fig. 2), whilst it was more evident in Tutopatch® grafts. Further collection of longitudinal data will hopefully allow us to evaluate further whether the trend for differences between Tutopatch® and Tutoplast® will continue, and whether both patch grafts undergo further thinning over time.

Our study has several limitations. With 50 patients and a 12-month follow-up, late tube exposures may have been missed [20]. However, we observed a faster and more extensive thinning of Tutopatch® compared to Tutoplast®. We only analysed two graft materials, though many exist with varying outcomes. A larger randomised trial could provide broader comparisons. De Luna et al. estimated that 435 patients are needed for 80% power to detect differences in tube exposure rates [5]. A multicentre study pooling data from various glaucoma centres could optimise treatment. Lastly, we focused only on PGI, limiting comparisons between different GDI.

## Conclusions

AS-OCT is an effective and reproducible modality to evaluate patch grafts after glaucoma drainage device surgery and monitor their thinning rate. It could potentially help identify patients at risk for implant exposure who might benefit from earlier surgical revision. When comparing Tutopatch® with Tutoplast®, the latter seems more durable and associated with less tube exposures, with a slower rate of thickness reduction. Future studies comparing other patch materials would be desirable.

## Summary

### What was known before:

- Anterior segment optical coherence tomography (AS-OCT) can be used to image filtering blebs and patch materials after PAUL® glaucoma implant surgery.

### What this study adds:

- AS-OCT can be used to quantify different patch grafts and their characteristics.
- Tutoplast® fascia lata seems to be superior to Tutopatch® pericardium to prevent tube erosions after PAUL® glaucoma implant surgery.

## Supplementary information

Bland-Altman plots of intra-rater reliability and inter-rater reliability analyses.

Pre- and post-operative data and patch graft measurements for Tutopatch® and Tutoplast® at 3 and 6 months.

Comparison of patients with Tutoplast® and Tutopatch®-patients with and without tube exposure at 3 and 6 months.
