# Supplementary material for: Comparing the use of Tutopatch® pericardium with Tutoplast® fascia lata in the context of PAUL® glaucoma implant surgery: an anterior segment OCT study
Source: Eye (Lond). 2025 Sep 23;39(16):2998–3004. doi: 10.1038/s41433-025-04018-3 (PMC12583483; doi:10.1038/s41433-025-04018-3)
Supplement: Supplementary file 1 — Bland-Altman plots of intra-rater reliability and inter-rater reliability analyses. [file 41433_2025_4018_MOESM1_ESM.docx]

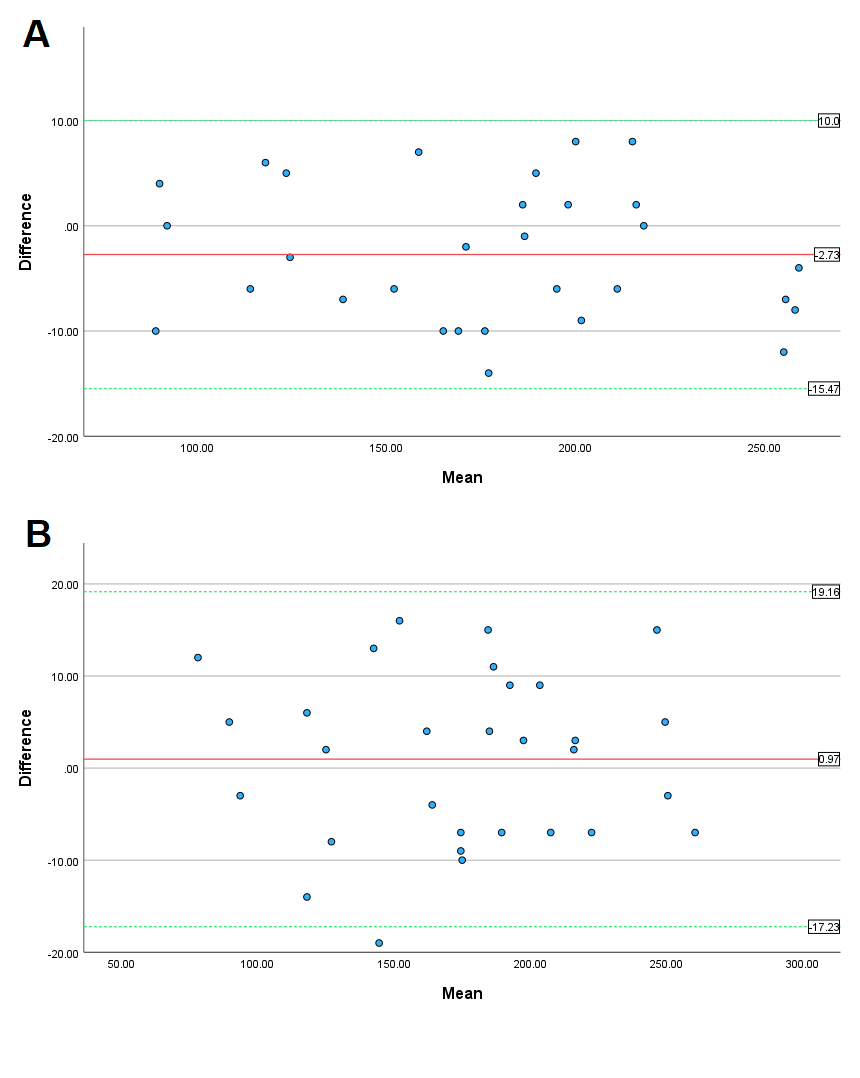


**Suppl. 1:** Bland-Altman plots of intra-rater reliability (A) and inter-rater reliability (B) analyses showing a very good reliability of the used AS-OCT imaging technique and protocol.
