# Supplementary material for: Comparing the use of Tutopatch® pericardium with Tutoplast® fascia lata in the context of PAUL® glaucoma implant surgery: an anterior segment OCT study
Source: Eye (Lond). 2025 Sep 23;39(16):2998–3004. doi: 10.1038/s41433-025-04018-3 (PMC12583483; doi:10.1038/s41433-025-04018-3)
Supplement: Supplementary file 2 — Pre- and post-operative data and patch graft measurements for Tutopatch® and Tutoplast® at 3 and 6 months. [file 41433_2025_4018_MOESM2_ESM.docx]

| **Variable** | **Tutopatch® (n = 26)** | **Tutoplast® (n = 24)** |
| --- | --- | --- |
| 3 months after surgery: |  |  |
| Mean conjunctival thickness (µm) | 204 ± 82 | 276 ± 115 |
| Mean patch material thickness above the tube (µm) | 992 ± 433 | 1408 ± 282 |
| Mean patch material thickness superior of the tube (µm) | 1101 ± 327 | 1595 ± 342 |
| Mean patch material thickness inferior of the tube (µm) | 1106 ± 323 | 1638 ± 335 |
| Mean overall patch material thickness (µm) | 1066 ± 353 | 1547 ± 309 |
| Fluid layer between the patch material layers (yes \| no) | 8 \| 18 | 7 \| 17 |
| Patch material exceeding the limbus (yes \| no) | 7 \| 19 | 15 \| 9 |
|  |  |  |
| 6 months after surgery: |  |  |
| Mean conjunctival thickness (µm) | 187 ± 122 | 251 ± 83 |
| Mean patch material thickness above the tube (µm) | 717 ± 545 | 1407 ± 239 |
| Mean patch material thickness superior of the tube (µm) | 879 ± 478 | 1570 ± 222 |
| Mean patch material thickness inferior of the tube (µm) | 878 ± 470 | 1654 ± 258 |
| Mean overall patch material thickness (µm) | 825 ± 471 | 1543 ± 226 |
| Fluid layer between the patch material layers (yes \| no) | 1 \| 25 | 5 \| 19 |
| Patch material exceeding the limbus (yes \| no) | 2 \| 24 | 4 \| 20 |

**Suppl. 2:** Pre- and post-operative data and patch graft measurements for Tutopatch^®^ and Tutoplast^®^ at 3 and 6 months.
