# Supplementary material for: Comparing the use of Tutopatch® pericardium with Tutoplast® fascia lata in the context of PAUL® glaucoma implant surgery: an anterior segment OCT study
Source: Eye (Lond). 2025 Sep 23;39(16):2998–3004. doi: 10.1038/s41433-025-04018-3 (PMC12583483; doi:10.1038/s41433-025-04018-3)
Supplement: Supplementary file 3 — Comparison of patients with Tutoplast® and Tutopatch®-patients with and without tube exposure at 3 and 6 months. [file 41433_2025_4018_MOESM3_ESM.docx]

| **Variable** | **Tutopatch® - No tube exposure**  **(n = 21)** | **Tutopatch® - Tube exposure**  **(n = 5)** | **Tutoplast®**  **(n = 24)** | **P-value** |
| --- | --- | --- | --- | --- |
| 3 months after surgery: | | | | |
| Conjunctival thickness (µm) | 226 ± 74 | 113 ± 46 | 276 ± 115 | *NTE/TE:* 0.002^b^  *Patch/Plast:*  0.0122^b^ |
| Mean patch  material thickness above  the tube (µm) | 1094 ± 382 | 564 ± 398 | 2408 ± 283 | *NTE/TE:* 0.013^b^  *Patch/Plast:*  0.0003^b^ |
| Mean overall  patch material  thickness (µm) | 1153 ± 318 | 704 ± 260 | 1547 ± 309 | *NTE/TE:* 0.012^b^  *Patch/Plast:*  < 0.0001^b^ |
|  | | | | |
| 6 months after surgery: | | | | |
| Conjunctival thickness (µm) | 223 ± 107 | 36 ± 34 | 251 ± 83 | *NTE/TE:* < 0.001^b^  *Patch/Plast:*  0.0073^b^ |
| Mean patch  material  thickness above  the tube (µm) | 849 ± 518 | 166 ± 210 | 1407 ± 239 | *NTE/TE:* 0.005^b^  *Patch/Plast:*  < 0.0001^b^ |
| Mean overall  patch material  thickness (µm) | 916 ± 446 | 440 ± 405 | 1543 ± 226 | *NTE/TE:* 0.067^b^  *Patch/Plast:*  < 0.0001^b^ |

NTE = ‘no tube exposure’, TE = ‘tube exposure’

^b^ *Mann-Whitney U test*

**Suppl. 3:** Group statistics and comparison of patients with Tutoplast^®^ and Tutopatch^®^-patients with and without tube exposure at 3 and 6 months.
